# Tumor-Associated Macrophages in Multiple Myeloma: Key Role in Disease Biology and Potential Therapeutic Implications

**DOI:** 10.3390/curroncol30070455

**Published:** 2023-06-25

**Authors:** Emanuele Cencini, Anna Sicuranza, Sara Ciofini, Alberto Fabbri, Monica Bocchia, Alessandro Gozzetti

**Affiliations:** Unit of Hematology, Azienda Ospedaliera Universitaria Senese, University of Siena, 53100 Siena, Italy; sicuranza4@unisi.it (A.S.); sara.ciofini@ao-siena.toscana.it (S.C.); fabbri7@unisi.it (A.F.); gozzetti@unisi.it (A.G.)

**Keywords:** multiple myeloma, tumor-associated macrophages, tumor microenvironment, drug resistance, prognosis

## Abstract

Multiple myeloma (MM) is characterized by multiple relapse and, despite the introduction of novel therapies, the disease becomes ultimately drug-resistant. The tumor microenvironment (TME) within the bone marrow niche includes dendritic cells, T-cytotoxic, T-helper, reactive B-lymphoid cells and macrophages, with a complex cross-talk between these cells and the MM tumor cells. Tumor-associated macrophages (TAM) have an important role in the MM pathogenesis, since they could promote plasma cells proliferation and angiogenesis, further supporting MM immune evasion and progression. TAM are polarized towards M1 (classically activated, antitumor activity) and M2 (alternatively activated, pro-tumor activity) subtypes. Many studies demonstrated a correlation between TAM, disease progression, drug-resistance and reduced survival in lymphoproliferative neoplasms, including MM. MM plasma cells in vitro could favor an M2 TAM polarization. Moreover, a possible correlation between the pro-tumor effect of M2 TAM and a reduced sensitivity to proteasome inhibitors and immunomodulatory drugs was hypothesized. Several clinical studies confirmed CD68/CD163 double-positive M2 TAM were associated with increased microvessel density, chemoresistance and reduced survival, independently of the MM stage. This review provided an overview of the biology and clinical relevance of TAM in MM, as well as a comprehensive evaluation of a potential TAM-targeted immunotherapy.

## 1. Introduction

Multiple myeloma (MM) represents a frequent hematological disease, characterized by a significant survival improvement during last years, thanks to the availability of novel agents, including proteasome inhibitors (bortezomib, carfilzomib), monoclonal antibodies (daratumumab, isatuximab, elotuzumab) and immunomodulatory drugs (thalidomide, lenalidomide, pomalidomide) [1,2,3,4]. However, a significant proportion of patients experience refractory disease or relapse (R/R) after an initial response [1,2,3,4,5]. Cytogenetic analysis can help to better stratify MM cases at diagnosis and/or at relapse into distinct risk categories with different prognosis [6]. Unfortunately, the presence of cytogenetic abnormalities does not fully explain the frequently observed drug resistance in MM cases [7,8]. The acquired drug resistance represents the leading cause of R/R disease and it can ultimately reduce progression-free survival (PFS) and overall survival (OS) [9,10,11,12].

**Figure 1:**
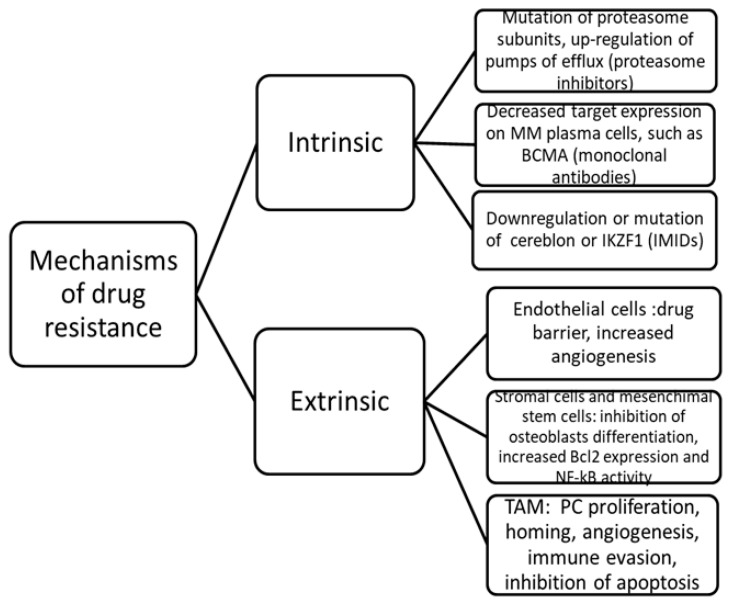
Principal intrinsic and extrinsic mechanisms of drug resistance in multiple myeloma. MM, multiple myeloma; BCMA, B-cell maturation antigen; IKZF1, Ikaros; IMIDs, immunomodulatory drugs; Bcl, B-cell lymphoma; NF-kB, nuclear factor-kB; TAM, tumor-associated macrophages; PC, plasma cells.

The multiple intrinsic and extrinsic mechanisms of drug resistance include the inhibition of drug entrance into neoplastic plasma cells, a drug-efflux pump that reduces drug activity, a mutation in the gene encoding the drug’s target protein and a drug inactivation due to bone marrow (BM) stromal cells which constitute the tumor microenvironment (TME), as illustrated in Figure 1 [13,14,15,16,17,18,19,20].

The TME in the BM includes hematopoietic stem cells, endothelial cells, osteoclasts, osteoblasts, extracellular matrix proteins, mesenchymal stem cells (MSC) and tumor-associated macrophages (TAM) [21,22,23,24,25,26,27]. Stromal cells can contribute to drug resistance by the secretion of cytokines that promote the production of antiapoptotic proteins of the B-cell lymphoma (Bcl)-2 family [22,28]. MSC in association with TAM and endothelial cells can foster an immunosuppressive TME and form a “vascular niche”, which can protect MM cells from antineoplastic drugs, such as bortezomib [22,26,29,30].

Macrophages can release pro-inflammatory cytokines, promote the recruitment of leukocytes to the site of inflammation and give a contribution to the tissue reparation and phagocytosis of foreign antigens, such as neoplastic antigens [31,32,33]. The phagocytic activity of macrophages against tumor cells can be directly performed through the binding to antibodies located on the tumor cells surface [32,33,34]. After phagocytosis, macrophages perform an antigen-presenting cell (APC) function, by the exposure on their surface of tumor antigen together with class II major histocompatibility complex (MHC II), thus permitting its recognition by T-lymphocytes [34,35]. Subsequent secondary signals include the engagement of costimulatory molecules, with CD40 ligand (CD40L) expressed by T cells engaging CD40 expressed by macrophages. After CD40 activation, macrophages release pro-inflammatory cytokines such as tumor necrosis factor (TNF)α and increase their expression of MHC II, which may in turn stimulate the anti-neoplastic activity of T-cell [36].

However, an elevated macrophage number, as frequently reported in hematologic malignancies, could also contribute to tumor progression by multiple mechanisms, including angiogenesis, the reduction in CD8 T-cell proliferation, the recruitment of T-regulatory cells (T-regs) and the inhibition of apoptosis [37,38,39,40,41].

**Figure 2:**
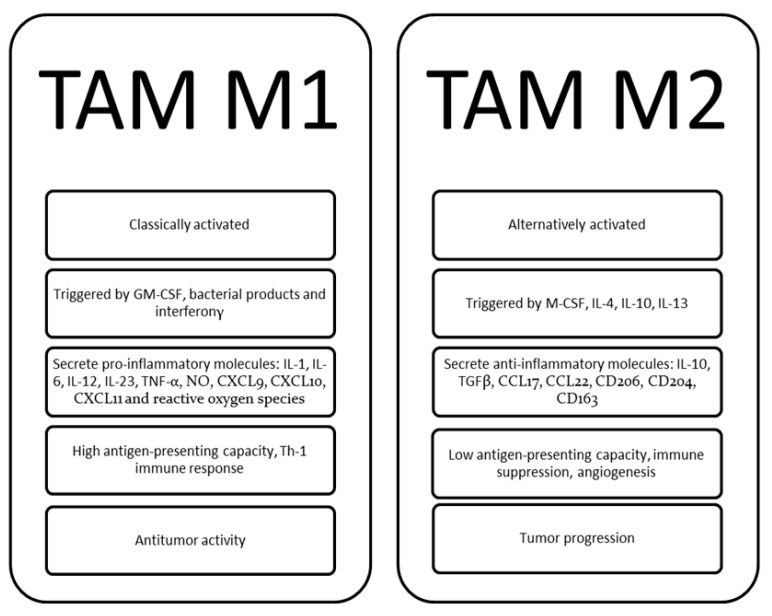
Tumor-associated macrophages polarization and functions of the main subtypes M1 (classically activated) and M2 (alternatively activated). TAM, tumor-associated macrophages; GM-CSF, granulocyte and monocyte colony stimulating factor, IL, interleukin; TNF, tumor necrosis factor, NO, nitric oxide; CXCL, chemokine ligands with C-X3-C motif; Th, T helper; M-CSF, monocyte colony stimulating factor; CCL, chemokine ligands with a C-C motif; TGF, tumor growth factor.

The so-called TAM, as “bad guys”, are characterized by a complex interaction with malignant cells for patients with non-Hodgkin lymphoma (NHL), Hodgkin lymphoma (HL), acute myeloid leukemia (AML), acute lymphoblastic leukemia (ALL), chronic lymphocytic leukemia (CLL) and MM [42,43,44,45,46,47,48,49,50,51,52,53,54,55,56,57]. TAM are identifiable by the CD68 marker but are further characterized by remarkable plasticity and were divided in the current classification into M1 (classically activated) and M2 (alternatively activated) [58,59,60]. The M1 TAM subtype could provoke a Th-1 immune response and play an antitumor effect, while M2 TAM have a low antigen-presenting capacity and could promote tumor progression by inducing immunosuppression and angiogenesis, as illustrated in Figure 2 [60,61]. From a biological point of view, mature macrophages in humans are identifiable by some CD markers, including CD11b, CD11c, CD14, CD16, CD68, CD115, CD312 [36]. Interestingly, TAM M1 showed an elevated expression of CD38, CD40, CD64, CD80, CD86, while TAM M2 express high levels of CD163, CD204 and CD206 [36].

We suggest TAM represent a subpopulation of macrophages located in the tumor site, which is strongly influenced by cancer cells and TME. TAM originate from circulating monocytes after recruitment at tumor site by cancer cells and progressively acquire pro-tumor properties, making themselves similar to M2 macrophages that are present in the site of injury upon removal of damaged tissue (the so-called “wound that does not heal”) [36,60].

In this field, during tissue reparation, there is a transition between classically activated M1 and alternatively activated M2 macrophages, which, in turn, coordinate the proliferation of cell subtypes useful for wound healing, such as vascular endothelial cells and fibroblasts [60]. Tumor cells showed the capability to manipulate immune response with the aim of creating a pro-wound healing, anti-inflammatory macrophage phenotype. A possible explanation is that immune response against cancer is not effective, due to the immune-editing hypothesis, in which the inflammatory response could force tumor cells to reduce the expression of antigenic proteins and to limit the subsequent presentation of these antigens to immune cells of the host [60]. According to this hypothesis, there is a limited macrophage responsiveness to neoplastic antigens and a reduced transition towards an M1 subtype. In addition, through the equilibrium/escape immune-editing processes and/or the capability of neoplastic cells to provide similar cues to those promoting a pro-wound healing response, there is an overall promotion of an M2 phenotype [60].

Specifically, the M1 subtype is activated by granulocyte-monocyte colony-stimulating factor (GM-CSF), interferon (IFN)-γ and bacterial products, while M2 activation is triggered by interleukin (IL)-4, IL-10, IL-13 [33,58,59,60,61]. Furthermore, M1 TAM secrete molecules with pro-inflammatory activity, such as IL-1, IL-6, IL-12, IL-23, TNF-α, nitric oxide (NO), chemokine ligands with a C-X3-C motif (CXCL)9, CXCL10 and CXCL11 [33,58,59,60,61]. Conversely, M2 TAM express anti-inflammatory molecules, including IL-10, tumor growth factor (TGF)-β, chemokine ligands with a C-C motif (CCL)17, CCL18, CCL22, class A scavenger receptor (CD204), mannose receptor C type 1 (CD206) and hemoglobin scavenger receptor (CD163) [58,59,60,61].

However, the separation between M1/M2 subtypes was developed more than 20 years ago and it could represent an oversimplification of a broader spectrum, including at least five subsets: M1, M2a, M2b, M2c and M2d. M2a was induced by IL-4 and/or IL-13 and showed a primary anti-inflammatory and pro-wound healing function. M2b was induced by IL-1b and demonstrated an immuno-regulatory role. Conversely, M2c were induced by IL-10 and showed an increased expression of tissue remodeling and immune suppressive markers. Finally, M2d subtype could be induced by IL-6 and could express angiogenic markers [60].

To exert their action, TAM have to be recruited within TME; the recruitment is mediated by chemokines, including CCL2, CCL5, CCL7 and CXCL1, vascular endothelial growth factor (VEGF) and M-CSF [62,63,64,65,66]. Afterwards, at the tumor site, TAM have to be polarized into a tumor-promoting M2 phenotype, thanks to a complex interaction with stromal cells and malignant cells, such as MM plasma cells [26,67,68,69,70,71]. Cancer cells could influence TAM through the interaction between CD47 on tumor cells and the signal regulatory protein (SIRP)α; this pathway generates a “do not eat me signal”, that could protect tumor cells from macrophage-mediated phagocytosis [72,73,74]. Once polarized, TAM could, in turn, influence neoplastic progression, drug resistance and metastasis, by producing matrix remodeling molecules and reducing both innate and adaptive immune cells function [67,68,69,70,71].

This review illustrates the manuscripts associated with the influence of TAM, with a particular focus on M2 subtype, in MM pathophysiology, proliferation, disease progression and drug resistance. Finally, we will also provide a summary of the possible use of TAM as a therapeutic target.

## 2. Materials and Methods

We performed a computerized search in MEDLINE to find publications as full-text, written in English, focused on the relationship between TAM and MM. The key terms of our search included “tumor-associated macrophages OR TAM OR M1 macrophages OR M2 macrophages OR CD68 OR CD163 OR CD204 OR CD206 AND multiple myeloma.”

We also searched in the reference list of selected articles to perform a more comprehensive research. We excluded conference abstract and case reports, but we included retrospective studies. For each preclinical and clinical study, we extrapolated: methods of TAM determination, TAM markers, patient number (if clinical study), treatment schedule and the relationship between TAM, drug resistance and disease outcome, especially PFS and OS.

## 3. TAM Role in the Pathophysiology and Progression of MM

TAM play a key role in MM pathophysiology, since they represent up to 10% of the BM cells of MM cases and could support disease progression, immune evasion, angiogenesis and drug resistance with multiple mechanisms, as represented in Table 1 [22,23,24,25,26,27].

### 3.1. TAM Accumulation

#### 3.1.1. Mouse Model Studies

Immunohistochemical (IHC) studies showed an inconsistent evidence that total TAM number was increased in the BM of MM-bearing mice, if compared to monoclonal gammopathy of uncertain significance (MGUS) [75]. Conversely, a significant increase was demonstrated by studies using flow cytometry and macrophages were physically associated with clonal PC [76]. In vitro, MM plasma cells could favor an M2 polarization by upregulating the CD206 expression of cocultured macrophages [77].

#### 3.1.2. Human Studies

MM cells are known to secrete chemotactic factors that could influence the monocytes migration into the BM [78,79,80]. In addition, TAM and BM stromal cells could, in turn, recruit circulating monocytes from peripheral blood into the TME [78,79,80]. A possible explanation was that there was a selective increase in M2 TAM in the BM of MM cases, suggesting the capability of MM cells to drive TAM polarization towards an M2 subtype [81,82,83]. Moreover, several studies confirmed TAM number was increased for MM patients with aggressive disease [81,82,83,84]. Consistently, the total BM number of CD206-positive M2 TAM was increased for MM cases with active disease, if compared to healthy controls or MGUS cases, while the M1 TAM number was not significantly different [81–8cencini4]. A growing relevance for MM pathogenesis was recognized to the JAK/STAT pathway activation in both TAM and MM cells [85,86,87]. TAM could support MM cells survival through the activation of IL-6/JAK/STAT3 pathway, as demonstrated in a coculture of TAM together with 5T33MM MM cells [85].

### 3.2. TAM and PC Migration, Homing and Proliferation

#### 3.2.1. Mouse Model Studies

A cornerstone for MM progression is represented by the initial homing and subsequent establishment within the BM of malignant PC [88]. In vitro, Opperman and colleagues demonstrated an increased, dose-dependent trans-endothelial migration of murine MM PC (line 5TGM1) towards a medium conditioned with BM-derived macrophages [88]. Due to the TAM production of insulin-like growth factor (IGF)-1 (with an elevated IGF-1 mRNA level expressed by BM-derived macrophages), a clodronate-liposome-mediated TAM depletion hindered MM development both in vitro and in vivo by reducing the migration and homing of 5TGM1 MM cells within the BM [88].

#### 3.2.2. Human Studies

Chemotactic molecules produced by TAM, such as IL-8, CCL2, CCL3 and (IGF)-1, play a relevant role in PC migration and homing [89,90,91,92,93]. In addition, in vitro and ex vivo studies demonstrated that the presence of macrophages in co-culture could increase the PC growth rate and proliferation through IL-6, IL-10 and IGF-1 secretion, together with the reduced production of IL-12 and TNF-α [94,95,96].

The role of IL-6 as PC growth factor was confirmed in a study in which the presence of IL-6 blocking antibody could reduce the proliferation index of co-cultured MM cells; interestingly, this effect was more pronounced in the presence of MSC and/or TAM [93]. Furthermore, IL-6 leads to an increased IL-10 production, which, in turn, could promote MM cells proliferation and survival [94,97]. IL-6 could stimulate c-Myc expression in MM cells due to an enhancement of c-Myc translation and it is a well known adverse prognostic factor in MM; however, it is produced by M1 TAM in addition to other cytokines [98,99]. A possible explanation is that IL-6 is a pleiotropic cytokine, with conflicting data about its role in inflammation [100,101]. Even if multiple studies demonstrated IL-6 was a pro-inflammatory cytokine in various settings, it could also promote the alternative activation of macrophages and exert an anti-inflammatory effect [100,101]. One possibility is that IL-6 could play a pro-inflammatory role in acute inflammation and an anti-inflammatory role at lower levels and/or in different cell subtypes. Finally, the binary division between M1 and M2 TAM is probably oversimplified and a broader spectrum of subtypes exists in humans [60,100,101].

### 3.3. TAM and Angiogenesis

#### 3.3.1. Mouse Model Studies

Access to vascular structures by proximity is critical to guarantee to MM cells an adequate supply of oxygen and nutrients. A growing evidence suggests TAM, within BM TME, could play a pro-angiogenic role through vascular mimicry and VEGF production, synergizing with the angiogenic properties of malignant PC [76]. In a mouse model, an increased microvessel density (MVD) and an elevated production of pro-angiogenic cytokines were associated with the presence of CD206-positive TAM [76]. In another mouse models, BMI1, a polycomb-group protein, could modulate the pro-MM function of TAM, which expressed higher BMI1 levels, if compared to normal macrophages [102]. In a BMI1 knockout mouse, an inferior TAM proliferation and a reduced expression of angiogenic molecules was reported [102]. In addition, the accumulation of Tie2^+^ pro-angiogenic macrophages was associated with increased angiogenesis and disease progression in the Vk*Myc murine MM model [76,103].

In the MM nude mouse subcutaneous xenograft model, TAM removal from the TME by using clodronate-liposome hindered tumor growth [88]. Moreover, M1 and M2 TAM could inhibit and promote tumor growth, respectively. The association of clodronate-liposome and the VEGF small interference(si)RNA, a molecule which depleted VEGF, significantly reduced tumor volume if compared to the administration of clodronate-liposome as single-agent [88].

#### 3.3.2. Human Studies

In several reports, during progression from MGUS to MM, M2 TAM could direct angiogenesis through its expression and production of VEGF, the main angiogenic player [104,105,106,107]. In addition, if TAM were exposed in vitro to VEGF, they could acquire endothelial cells markers and generate capillary-like vessels, a process named vasculogenic mimicry [83]. Notably, macrophages could maintain their lineage markers, such as CD14 and CD68, without an endothelial trans-differentiation [83,107].

In another study, TAM showed the capability to secrete IL-10, that could induce both PC proliferation and angiogenesis [108].

TAM synthesize several angiogenic factors other than VEGF, such as fibroblast growth factor (FGF)2, inducible nitric oxide synthase (iNOS), IL-8 and TNFα [106,107]. The above-mentioned study by Calcinotto and colleagues included a part in which human macrophages were able to stimulate the migration of endothelial cells and increase their capillarogenesis, similarly to what happens following VEGF or FGF2 stimulation in vitro [76]. Finally, micro (mi)RNA were also investigated as potential contributors to MM pathogenesis; in this field, exosome-derived miR-let-7c and miR-214 were involved in a recent study in both M2 TAM polarization and angiogenesis promotion within the BM TME [109].

Overall, these findings indicate TAM as a relevant component of the MM-associated pro-angiogenic network.

### 3.4. TAM and Immunosuppression

#### 3.4.1. Mouse Model Studies

In vivo, the administration of the anti CSF-1 receptor antibody CS7 significantly reduced the MM tumor burden, while the in vitro administration of low-dose CS7 could contribute to TAM polarization towards an M1 subtype [110]. M1 TAM showed an improved antigen-presenting capacity and the ability to enhance a cytotoxic CD4^+^ T cell response, further supporting the hypothesis that M2 TAM, conversely, play a relevant role in the decreased T cell activation and resulting immunosuppression reported in MM, ultimately leading to disease progression [110].

#### 3.4.2. Human Studies

Macrophages, as a member of the innate immune system, could directly influence the development of an immunosuppressive TME in MM [27,33]. TAM demonstrated the capability to suppress immune responses in MM by multiple mechanisms, including the inhibition of cytotoxic T cell response and the overexpression of immune checkpoint proteins [27,85,111,112]. TAM located within TME lost the ability to present antigens, engulf neoplastic cells and stimulate an adaptive immune response [22,23,24].

Notably, the expression on MM CD138^+^ PC of the immune check protein CD47, which inhibits the macrophages-mediated phagocytosis of neoplastic cells by binding to SIRPα (the above mentioned “do not eat me signal”), was very elevated and was associated with disease stage [73,74]. In vitro, an anti-CD47 antibody significantly improved the ability of macrophage to engulf MM cells [113,114]. In addition, TAM could drive immune tolerance, due to co-culture with TAM could increase programmed death receptor 1(PD1) expression on CD8^+^ T cells and programmed death receptor ligand 1 (PD-L1) expression on MM cells [102,110].

An in vitro suppression of T cell proliferation was obtained when MM cells were co-cultured with macrophages, further confirming MM cells could cross-talk with macrophages and polarize TAM towards an M2 immunosuppressive phenotype [85]. TAM could downregulate relevant T cell factors (such as gradzyme and IFN γ) and produce IL-10, which, in turn, inhibits production of inflammatory cytokines and limits cytotoxic T cell functions [77,94].

Another recently discovered mechanism is represented by the production of IL-32 by MM cells, which, in turn, increased production by TAM of indoleamine 2,3-dioxygenase (IDO), a molecule with a well-known inhibitory effect on T cells [115].

### 3.5. TAM and Drug Resistance

The tendency to develop drug resistance towards anti-MM drugs represents an unmet medical need for MM patients and a relevant role for TME, including TAM, was demonstrated during the last few years [12,27,33,116].

The resistance to the alkylating agent melphalan was mediated by the interaction between intracellular adhesion molecule-1 (ICAM-1) and P-selectin glycoprotein ligand-1 (PSGL-1) on MM cells and P-selectin and CD18 on TAM [117,118]. These bonds induce drug resistance through the activation of the Src, Erk1/2 kinase and c-myc pathway [117,118]. These findings were subsequently confirmed and the viability of MM cell lines treated with dexamethasone, bortezomib or lenalidomide was significantly increased if co-cultured with TAM; interestingly, these effects were mediated by M2 TAM, but not M1 TAM [118].

TAM could enable drug resistance towards bortezomib through the production of IL-1β, which in turn increased the number of MM-initiating cells [119]. In addition, when under the influence of MM cells, TAM could produce B-cell activating factor (BAFF), which impaired bortezomib-mediated apoptosis through the activation of NF-kB pathway [120]. Another relevant molecule is represented by CCL2, a chemokine with the ability to recruit TAM, trigger their polarization towards an immunosuppressive M2 subtype and stimulate TAM to express the monocyte chemoattractant protein-1-induced protein (MCPIP1), which could protect MM cells from bortezomib-mediated apoptosis [121].

Total TAM, determined as CD68-positive macrophages, were shown to inhibit in a pre-clinical model drug-induced apo ptosis by caspase 3 and poly-ADP ribose polymerase (PARP) cleavage [117,118]. Recently, JAK inhibition was shown to revert M2 TAM polarization and overcome the resistance to lenalidomide of MM cells, which occurred when MM cells were co-cultured with TAM [122].

## 4. Clinical Studies of TAM in MM

Several studies confirmed the prognostic relevance of TAM for MM patients, especially CD68/CD163 double-positive M2 TAM, as illustrated in Table 2. Overall, TAM were associated with an aggressive disease course and reduced survival, independently of the MM stage [119,123,124,125,126]. Patients with active disease presented with an increased number of CD206-positive M2 TAM within the BM, if compared to patients with MGUS or healthy subjects [81,123,124,125,126].

The difference of macrophage involvement in MM cases with different prognosis was initially demonstrated using CD68 as a single marker [33,123]. In a retrospective study, in which 68 MM patients were enrolled, TAM were determined with anti-CD68 and anti CD-163 antibodies. A detrimental effect on 6-y OS was demonstrated in a multivariate analysis for an elevated expression of both CD68-positive and CD163-positive TAM. Notably, an elevated CD163-positive TAM expression was associated with an increased microvessel density, further confirming M2 TAM were characterized by an adverse prognostic influence [123].

Subsequently, in 198 MM patients receiving bortezomib-based regimens, CD163 expression was assessed by IHC as macrophage marker with the used cut-off of >55/high power field [120]. MM patients with a high CD163-positive M2 TAM expression at diagnosis had a lower CR rate and reduced PFS and OS in multivariate analysis [124]. In addition, a significant association between an elevated level of soluble M2 TAM markers CD163 and CD206 and reduced OS was reported; conversely, a higher M1 density was associated with an improved OS [125,126]. Specifically, CD163 as a soluble protein was investigated in 104 blood samples and 17 BM samples of newly diagnosed MM patients. BM expression of CD163 was more elevated if compared to peripheral blood and was associated with higher international staging system (ISS) and other well-known prognostic factors [125,126]. The used cut-off of 1.8 mg/L was associated with adverse disease outcome, further suggesting the clinical relevance of CD163-positive TAM and the ability to influence MM progression [125,126].

The clinical impact of different TAM subtypes by IHC was illustrated in a study in which total TAM were considered as CD68-positive and subclassified as M1 TAM (iNOS-positive, classically activated) or M2 TAM (CD163-positive, alternatively activated) [81]. In this large cohort of 240 MM cases treated with proteasome inhibitors or immunomodulators, an inferior overall response rate (ORR) was reported for patients with elevated CD68-positive and CD163-positive TAM [81]. Interestingly, only an elevated CD163 expression was correlated with decreased PFS and OS. A new prognostic score was generated, in which CD163 and iNOS had a negative prognostic role and were combined with ISS [81].

These results were confirmed in another study, in which the authors reported low M1 TAM infiltration, assessed by the concurrent expression of CD68 and C-C chemokine receptor 2 (CCR2) by flow cytometry, was correlated with an unsatisfactory response to bortezomib [119]. In addition, CD163-positive M2 TAM could induce angiogenesis through the pro-angiogenic factor CD147, a matrix metalloproteinase inducer, in a spectrum of patients ranging from MGUS to relapsed/refractory (R/R) MM [84]. The used cut-off for CD163 as M2 TAM marker was 100 per core and the authors demonstrated high M2 expression was associated with reduced median OS for R/R MM (32 vs. 6 months, *p* = 0.02), further confirming an adverse prognostic influence for CD163-positive TAM [84].

As MM PC, but not normal PC, express CD47 to evade a macrophage-mediated phagocytosis, its expression was associated with disease progression from MGUS to MM [73,74]. The role of this checkpoint was supported by the evidence that CD16-positive monocytes were necessary to permit the killing of MM cells by an anti-CD47 antibody [127]. This CD47–SIRPα interaction could represent an interesting therapeutic target and anti-CD47 antibodies showed promising efficacy in preclinical models [127,128].

## 5. TAM as Possible Therapeutic Target for MM

The above-mentioned clinical relevance of TAM for MM patients led to translational research with the aim to discover the underlying mechanisms, as illustrated in Table 3.

The close interaction between TAM and MM cells could represent the basis to design targeted therapies and to overcome the well-known TAM-mediated drug resistance [129,130,131,132,133,134,135,136,137,138,139,140,141,142,143,144,145,146,147,148,149,150]. An elevated number of preclinical studies investigated TAM as a potential therapeutic target in a variety of neoplasms with the aim to overcome immunosuppressive barriers. In this field, different strategies include the blockade of monocyte recruitment, TAM depletion, TAM reprogramming into the immunostimulatory M1 subtype, molecular signaling modifications (such as the inhibition of immunosuppressive molecules produced by TAM and CD47/SIRPα checkpoint) and the reversal of drug resistance by targeting the cross-talk between TAM and tumor cells [129,130,131,132,133,134,135,136,137,138,139,140,141,142,143,144,145,146,147,148,149,150].

**Table 3:** The role of tumor-associated macrophages (TAM) as treatment target in multiple myeloma.

Reference	Treatment	Mechanism of Action	Type of Study	Results
Sun et al. [26]	IL-10R blocking antibody	TAM reprogramming	In vitro, in vivo, ex vivo	Reduced MM cell proliferation. Overcame drug resistance to lenalidomide and dexamethasone
De Beule et al. [75]	JAK1/2 inhibitor AZD1480	Overcome drug resistance	In vitro 5T33MM murine model	MM cells killing, reduced tumor burden, resensitize to bortezomib
Beider et al. [85]	Anti-CXCR4 antibody	Reduced TAM recruitment	Human MM cells	Disruption of MM cells-TME interaction, reduced MM cells proliferation
Opperman et al. [88]	Clodronate -liposome	TAM depletion	In vivo mouse model	Abrogates MM establishment, reduced tumor burden
Zhang et al. [102]	BMI1 inhibitor PTC596	TAM depletion, antiangiogenic	In vivo mouse model	Reduced tumor burden, improved mice survival
Wang et al. [111]	Monoclonal antibody CS7 against CSF-1R	Reduced TAM recruitment and proliferation, TAM reprogramming	MM cells in vitro In vivo mouse models	Dose-dependent cell death, tumor-specific CD4^+^ T-cell response. Additive efficacy with bortezomib
Chen et al. [120]	BAFF inhibitor	Overcome drug resistance	Xenograft model	delayed tumor growth, resensitize to bortezomib
Chen et al. [122]	JAK1/2 inhibitor Ruxolitinib	TAM reprogramming	MM cells in vitro In vivo mouse models	Reduced tumor burden, resensitize to lenalidomide
Cucè et al. [130]	Trabectedin	Macrophage killing due to CCL2-CCR2 signaling axis inhibition, antiangiogenic	Human MM cells	Apoptosis trigger VEGF depletion NK cells upregulation
Vo et al. [133]	Lenalidomide	TAM depletion	In vivo mouse model	Reduced IL10 production, reduced tumor burden
Jensen et al. [136]	Agonistic anti-CD40 antibody	TAM reprogramming	In vivo mouse model	Reduced tumor burden, improved mice survival
Gutierrez-Gonzalez et al. [137]	GM-CSF and MIF blockade	TAM reprogramming	MM cells, patient samples, xenograft model	Increased cell death, reduced tumor burden in mice
Bonanno et al. [143]	IDO inhibitor	Reduced TAM-mediated immunosuppression	Patient cells	Reverted Tregs expansion, improved Th1 response
Rastgoo et al. [149]	Synthetic miR-155	Inhibition of CD47-SIRPα do not eat me signal	MM cell lines and patient samples	MM cells phagocytosys by macrophages, apoptosis induction. Resensitize to bortezomib
Veitonmäki et al. [150]	BI-505, antibody against ICAM-1	Targeting crosstalk TAM mm cells	MM cell lines, xenograft model	MM cell growth inhibition, reduced tumor burden

Abbreviations: TAM, tumor-associated macrophages; IHC, immunohistochemistry; PFS, progression-free survival; OS, overall survival; VAD, vincristine, doxorubicin, dexamethasone; MP, melphalan, prednisone; iNOS, inducible nitric oxide synthase; TD, thalidomide, dexamethasone, MPT, melphalan, prednisone, thalidomide.

### 5.1. TAM Recruitment

#### 5.1.1. Mouse Model Studies

An important pathway for TAM recruitment and differentiation is represented by the CSF-1R signaling [129]. CSF-1R inhibition was able to block TAM polarization in mouse models of T-acute lymphoblastic leukemia (ALL) and the association between a CSF-1R inhibitor and vincristine could increase survival of leukemic mice, if compared to vincristine as monotherapy [129]. In the MM mouse model, Wang and colleagues added the monoclonal antibody CS7 against murine CSF-1R to monocyte cultures and induced a reduced TAM recruitment and a dose-dependent cell death [111].

#### 5.1.2. Human Studies

Controlling monocyte recruitment to the TME could represent a promising mechanism to reduce TAM infiltration and it is regulated by citokines and chemokines produced by tumor cells and stromal cells. Since monocytes trafficking is regulated by the CCL2-CCR2 signaling axis, its blockade could reduce tumor proliferation in solid malignancies [130]. CCL2 showed the ability to influence macrophage homing towards the BM and its polarization in MM. In addition, CCL2 secreted by MM cells in the TME could promote the M2 TAM polarization via the JAK2-STAT3 pathway activation [130]. Trabectedin, a DNA-binding sea squirt-derived molecule, was investigated in human leukemic and MM cells, in which it could kill monocytes and macrophages and exert an antiangiogenic role through the inhibition of VEGF and CCL2 production [130]. Specifically, trabectedin could trigger cell cycle arrest and apoptosis in MM cell lines, together with VEGF depletion and NK cells upregulation. Finally, the inhibition of CCR2 due to a monoclonal antibody could reverse this protective effect [130].

CXCL12-CXCR4 represents another mechanism to recruit macrophage towards the MM BM [131,132]. MM cells showed an elevated CXCL12 expression and CXCL12-CXCR4 axis could contribute to MM cell adhesion and migration. Furthermore, this axis could promote both monocyte recruitment and TAM differentiation towards an immunosuppressive M2 subtype with elevated CD206 expression [85]. The CXCR4 inhibition with a neutralizing antibody was able to suppress monocyte recruitment towards the BM [85,131,132].

### 5.2. TAM Depletion

The administration of clodronate-liposome demonstrated the ability to cause a global macrophage depletion in solid and hematologic malignancies [26,27,88]. In an MM mouse model, a pre-treatment with clodronate-liposome could significantly reduce tumor burden if compared to liposome-treated controls [88]. Interestingly, the inhibition of tumor development was achieved after a single infusion in mice with established MM [88].

In another in vivo study in a murine MM model, the therapeutic effects of CSF-1R blocking monoclonal antibodies were investigated. Specifically, CSF-1R blockade could inhibit the M2 TAM proliferation and differentiation and repolarize TAM towards an antitumor M1 subtype [111]. Notably, when co-cultured with TAM and MM cells, the anti CSF-1R antibody CS7 was able to inhibit MM growth in vivo by depleting TAM and inducing a cytotoxic CD4^+^ T-cell response [111]. Antitumor efficacy against established MM was improved when the CSF-1R blockade was associated with bortezomib or melphalan [111]. As murine monocytes and macrophages can be depleted by using a diphtheria toxin, an in vitro administration of this toxin was performed to achieve the ablation of macrophages co-cultured with MM cells [133]. After treatment, MM growth and progression were significantly reduced, further confirming that TAM play an important role and TAM depletion by CSF-1R inhibitors could represent a promising therapeutic strategy. Moreover, lenalidomide could contribute in mouse models to an M2 TAM depletion, together with a reduction in IL-10 release [133,134].

In addition, due to the BMI1 protein, released by MM cells, could promote TAM proliferation, angiogenesis and drug resistance, its inhibitor PTC596 was investigated in a murine MM model. This agent was able to reduced MM tumor burden and improve mice survival by depleting M2 TAM [101].

In our opinion, TAM depletion, especially by using biphosphonates, could represent a promising therapy for MM patients. In particular, biphosphonates are already used in clinical daily practice to prevent skeletal complications in MM cases [135]. These molecules could have an additional therapeutic role, due to their pro-apoptotic properties and the ability to reduce MVD, further suggesting a possible association with anti-VEGF molecules, such as lenalidomide [135].

### 5.3. TAM Reprogramming

#### 5.3.1. Mouse Model Studies

In the above-mentioned study, the potential therapeutic effect of an anti-CSF-1R monoclonal antibody was investigated. In vivo, CSF-1R blockade could inhibit MM growth by both depleting and polarizing TAM towards the M1 subtype [111].

In a preclinical study, the JAK1/2 inhibitor ruxolitinib demonstrated the ability to suppress M2 TAM through the reduction in the tribbles homolog 1 protein kinase expression [122]. In addition, ruxolitinib could increase M1 polarization in vitro or in MM xenograft models in vivo and demonstrated the ability to restore sensitivity to lenalidomide [122].

Another promising target is represented by CD40, a cell surface costimulatory protein expressed on antigen-presenting cells (APC) and necessary for their activation. Agonistic antibodies were able to stimulate innate and adaptive immune response in cancer patients. A preclinical study in MM observed a repolarizing effect on TAM after sequential CD40 activation together with Toll-like receptor (TLR) ligation [136].

#### 5.3.2. Human Studies

TAM reprogramming aims to reduce the immunosuppressive M2 subtype, while promoting the immunostimulatory M1 subtype and represents a promising research field for MM therapy [137]. Interestingly, it could prevent disease progression from MGUS to active MM by the reduction in angiogenesis. A prospective study of circulating chemokines and angiogenic markers showed a significant association with future progression for MGUS cases who presented with elevated baseline levels of epidermal growth factor (EGF), FGF and Ang-2 [138]. Due to M2 TAM playing a pro-angiogenic role, we suggest an increased angiogenesis could represent the best mechanism to explain disease progression and could be counterbalanced by TAM reprogramming towards an M1 subtype.

In a pivotal paper, M1 TAM and M2 TAM, when co-cultured with MM cells, could play an antitumor and a protumor role, respectively. A double treatment with GM-CSF, a pro-M1 cytokine, in association with an inhibitor of the pro-M2 cytokine macrophage migration inhibitory factor (MIF) was performed [137]. This combination achieved the best reprogramming response, at both gene and protein expression level, confirming the hypothesis that TAM could reacquire their antitumor M1 phenotype, in response to appropriate stimuli. Moreover, a significant treatment efficacy was reported, with cytotoxic effect and increased MM cell death [137].

Finally, a relevant role was discovered for the IL-10/IL-10R pathway. Due to IL-10 being released by MM cells and potentially polarizing TAM towards an M2 subtype, the inhibition of IL-10/IL-10R pathway by using an anti-IL-10R blocking antibody could reprogram TAM to lose the M2 phenotype [26]. The final result of this reprogramming was represented by the reduction of MM proliferation along with the restoration of sensitivity to lenalidomide and dexamethasone [26].

In our opinion, TAM reprogramming represents an interesting research field for MM patients. Unfortunately, the anti-CD40 agonistic monoclonal antibody mitazalimab showed only a modest activity in patients with solid neoplasms with moderate toxicity in a phase I study [139]. Conversely, ruxolitinib in association with methylprednisolone was investigated in R/R MM cases, including patients with high-risk cytogenetics, with an encouraging ORR of 31% and a median duration of response of 13.1 months [140]. We suggest ruxolitinib should be investigated in association with other drugs with clinical activity against MM, such as steroids and lenalidomide.

### 5.4. Restoration of T-Cell Response and Inhibition of CD47/SIRPα Don’t Eat Me Signal

Due to TAM significantly contributing to immunosuppression and reducing T-cell response, the restoration of an adequate immune response through targeting TAM represents an interesting strategy. Notably, the lack of effect of several drugs in MM could be due to their effect on TME [12,27]. Cladribine, a purine analog used to treat hairy cell leukemia, NHL and systemic mastocytosis with variable treatment modalities, showed an unsatisfactory efficacy when administered to MM patients [141,142]. A recent study demonstrated that in M1 TAM, cladribine reduced the phagocytic activity but did not influence unactivated cells [142]. MM cells mediate the production by TAM of indoleamine 2,3-dioxygenase (IDO), an immunosuppressive molecule, through binding to proteinase 3 (PR3) on TAM and final activation of STAT3 and NF-kB pathways. The inhibition of these pathways and/or PR3 blockade in TAM could reduce IDO production, which, in turn, restored CD4^+^ T-cell response and increased the production of anti-inflammatory citokines. Another study used an IDO inhibitor, called D,L-1methyl-tryptophan, in patient MM cells and reported the reversal of T-regs proliferation and the improvement of Th1 immune response [143].

Immune checkpoints represent inhibitory mechanisms frequently used by tumor cells to escape from recognition and killing by cells of the host immune system. Anti PD-1/PD-L1 monoclonal antibodies demonstrated high efficacy in HL treatment, but treatment results were disappointing in MM [110,144]. The PD-L1/PD-1 axis was investigated by flow cytometry in the BM samples of 141 patients, including MGUS, smoldering MM (SMM) and active MM, either newly diagnosed or R/R cases. PD-L1 expression on MM cells was more elevated in SMM and MM cases if compared to MGUS [145]. Even if a rationale for the association with the anti-CD38 antibody daratumumab exists, clinical benefit was minimal and clinical trials were terminated for administrative reasons [146].

Since hematological malignancies, including MM, express high CD47 levels, preclinical research demonstrated that an antibody-mediated blockade of CD47-SIRPα signaling could promote tumor cell death, phagocytosis and improve T-cell response [73,74,127,128]. Several antibodies and SIRPα fusion proteins were designed and are under investigation, such as Hu5F9-G4, CC-90002, TTI-621 and ALX-148 [147]. The main clinical study in this field is the phase I trial in which the Hu5F9-G4 antibody was investigated in 22 R/R NHL cases; ORR was 50%, with an encouraging CR rate of 36% [148].

In addition, miR-155 was expressed at low levels in drug resistant MM cells and could directly regulate CD47 through its 3′UTR. An overexpression of miR-155 could suppress CD47 expression on MM cells surface, leading to the phagocytosis of MM cells by macrophages and the induction of apoptosis through targeting TNF AIP8 in vitro and in vivo [149].

### 5.5. Targeting the Cross-Talk between TAM and MM Cells to Overcome Drug Resistance

#### 5.5.1. Mouse Model Studies

As previously illustrated, the JAK2 inhibitor ruxolitinib was able to reduce M2 TAM polarization in MM both in vitro and in vivo [75]. Interestingly, ruxolitinib could also downregulate CXCL12 and CXCR4 in MM cells, the expression of which is associated with drug resistance to lenalidomide. These findings could provide the rationale to investigate ruxolitinib in association with lenalidomide for R/R MM patients [75]. In addition, AZD1480, a potent and competitive JAK1/2 inhibitor, was investigated as a strategy to inhibit the JAK/STAT3 pathway, finally improving the caspase-3-mediated apoptosis of MM cells. AZD1480 was very effective in vitro and could abrogate the TAM-mediated MM cell survival by restoring sensitivity to bortezomib [75].

Another strategy to overcome the bortezomib resistance was to target BAFF by neutralizing antibodies. In a xenograft model, anti-BAFF antibody in association with bortezomib showed a significantly delayed tumor growth and progression if compared to bortezomib as single-agent [120].

#### 5.5.2. Human Studies

A growing evidence was reported to indicate that TAM could contribute to MM drug resistance to several agents, including bortezomib, melphalan and lenalidomide [12,26,27]. A few strategies were investigated to target the crosstalk between TAM and MM cells with the aim to overcome drug resistance.

ICAM-1 expressed on MM cells interacts with CD18 on TAM surface and plays a relevant role to confer drug resistance to MM cells [150]. BI-505, a monoclonal antibody against ICAM-1, in preclinical studies inhibited cell growth and bone damage; in vivo, its efficacy against MM was macrophage-dependent. Unfortunately, a phase I dose-escalation study demonstrated limited efficacy in R/R MM, even if toxicity profile was satisfactory [150]. Finally, in an above-mentioned preclinical study, miR-155 overexpression could suppress CD47 on MM cells and re-sensitize drug resistant MM cell lines to bortezomib, further confirming the inhibition of CD47-SIRPα signaling could represent a promising treatment strategy to counteract the immunosuppressive TME of MM within the BM [149].

## 6. The Role of TAM in the Era of Novel Agents

Overall, there are limited available data about the role of TME, including TAM, in the natural history of MM. The adverse ISS stage, together with high-risk cytogenetics, was associated with distinct immune profiles in a cohort of MM cases receiving bortezomib, lenalidomide and dexamethasone (VRD) [151]. In this study, a unique profile characterized by higher T cells, with reduced erythroblasts and TAM, could identify patients who achieve a CR after receiving VRD as induction therapy. Remarkably, there was a skewed ratio towards M2 subtype in patients who achieve worse responses, and an increased TAM expression was demonstrated for patients with positive minimal residual disease (MRD+), if compared to MRD- MM cases [151].

Lenalidomide demonstrated the ability to drive TAM towards an immunostimulatory M1 subtype through the cereblon-CRL4 E3 ligase to degrade by ubiquitination the transcription factor IKAROS family zinc finger 1 (IKZF1) [152]. Lenalidomide could also counteract the pro-angiogenic properties of M2 TAM through a negative modulation of VEGF [153]. In addition, as mentioned above, lenalidomide could contribute in mouse models to an M2 TAM depletion, together with a reduction in IL-10 release [133]. Lenalidomide and pomalidomide demonstrated the ability to influence TME through the inhibition of myeloid-derived suppressor cells (MDSC), due to the cereblon-mediated downregulation of CCL5 and MIF in MM cells and induction of IFN regulatory factor 8, a relevant transcription factor for monocyte differentiation [154].

Due to preclinical studies demonstrated CD47 blockade could induce TAM activation, resulting in MM cells death, the anti CD47 magrolimab could be used as single-agent or in combination with other drugs in MM. Magrolimab in association with commonly used anti MM therapies is under investigation in an ongoing phase II study [155].

In recent years, the introduction of anti CD38 monoclonal antibodies daratumumab and isatuximab dramatically changed MM treatment landscape [1,2,156,157]. These drugs have multiple mechanisms of action, including PC killing through macrophage-mediated antibody-dependent cellular phagocytosis (ADCP) [156]. Unfortunately, a significant proportion of patients develop drug resistance during treatment, especially MM patients characterized by high cytogenetic risk [12,158]. Anti-CD38 antibodies were able to promote T-cell expansion and suppress T-regs, together with an increase in monocyte count in responders [159,160,161]. However, the investigation of TAM subtypes was not performed in these studies.

Finally, TAM influence in the response to antibody-drug conjugates, chimeric antigen receptor T cells (CAR-T) and Bi specific T-cell engagers is currently under investigation. In a recently published preclinical paper [162], RO7297089, a novel bispecific BCMA/CD16A-directed innate cell engager, demonstrated the ability to induce the lysis of BCMA-positive MM cells through multiple mechanisms, including retargeting of NK cell cytotoxicity and the activation of macrophage-mediated phagocytosis. Interestingly, the drug showed a favorable toxicity profile in vitro and in cynomolgous monkeys [162].

## 7. Conclusions

The treatment landscape of MM was revolutionized in recent years and the modulation of TME, including TAM, could represent a promising strategy to overcome MM drug resistance, in association with conventional and/or novel agents. We summarized the critical TAM influence in MM pathophysiology, disease progression, immunosuppression and drug resistance. Specifically, M2 TAM, which are characterized by immunosuppressive phenotype, were associated with reduced PFS and OS for MM patients. However, most of the available studies were preclinical and a great effort will be necessary to bring these findings from bench to bedside, with the aim to design safe and effective therapies for R/R MM cases.

Interestingly, a TAM-targeted treatment strategy could be associated with checkpoint inhibitors, CAR-T cells and/or Bi specific T-cell engagers, with the aim to reduce TAM-mediated immunosuppression.

In conclusion, TAM could represent an interesting target for future therapies, to counteract the frequently observed drug resistance in R/R MM cases, but many issues still need to be explored, including understanding the molecular mechanisms regulating cross talk between TAM, MM cells and other elements within TME. Finally, these promising results have to be validated in clinical studies, with the aim to give to each patients an increasingly personalized treatment strategy.

## Figures and Tables

**Table 1:** Current concepts about functional roles of TAM in regulating MM progression.

Event	Mechanisms	Main Effects
TAM Accumulation within TME	Chemotactic factors and M2 TAM polarization driven by MM PC, CD206 up-regulation on TAM, JAK/STAT activation.	Recruitment of circulating monocytes, MM cells growth and survival, aggressive disease.
PC migration, homing, proliferation	M2 TAM production of chemotactic molecules and PC growth factors	Increased proliferation index of MM cells
Angiogenesis	Vascular mimicry, VEGF, FGF production by M2 TAM	Generate capillary-like vessels, disease progression
Immunosuppression	Inhibition of cytotoxic T cell response, overexpression of immune checkpoint proteins	Reduced cellular immune response against MM cells
Drug resistance	Activation of the Src, Erk1/2 kinase and c-myc pathway. BAFF production. NF-kB pathway and IL-6/JAK/STAT3 pathway. TAM M2 polarization.	Increased MM cells viability. Impaired drug-mediated apoptosis

Abbreviations: TAM, tumor-associated macrophages; TME, tumor microenvironment; PC, plasma cells; MM, multiple myeloma; VEGF, vascular endothelial growth factor; FGF, fibroblast growth factor; BAFF, B-cell activating factor; IL, interleukin; JAK, Janus kinase.

**Table 2:** Clinical studies about prognostic role of tumor-associated macrophages (TAM) in multiple myeloma.

Reference	Number of Patients	TAM Marker	Technique	Treatment	Survival Correlation
Chen et al. [81]	240	CD68 CD163 iNOS	IHC	MP = 12, VAD = 37, TD/MPT = 161, bortezomib or lenalidomide = 30	Inferior PFS, OS
Panchabbai et al. [84]	141	CD163	IHC Flow cytometry	NR	Inferior OS
Beyar-Katz et al. [119]	34	CD68 CCR2	Flow cytometry	Bortezomib	Inferior OS
Suyani et al. [123]	68	CD68 CD163	IHC	VAD = 37, MP = 10, thalidomide = 11, bortezomib = 5, lenalidomide = 1, no treatment = 4	Inferior OS
Wang et al. [124]	198	CD163	IHC	Proteasome inhibitors	Inferior PFS, OS
Andersen et al. [125]	104	CD163	Soluble	High-dose therapy = 42 Chemotherapy = 62	Inferior OS
Andersen et al. [126]	104	CD206	Soluble	High-dose therapy = 42 Chemotherapy = 62	Inferior OS

Abbreviations: TAM, tumor-associated macrophages; IHC, immunohistochemistry; PFS, progression-free survival; OS, overall survival; VAD, vincristine, doxorubicin, dexamethasone; MP, melphalan, prednisone; iNOS, inducible nitric oxide synthase; TD, thalidomide, dexamethasone, MPT, melphalan, prednisone, thalidomide.
